# *In Vivo* Effects of Bradykinin B_2_ Receptor Agonists with Varying Susceptibility to Peptidases

**DOI:** 10.3389/fphar.2015.00306

**Published:** 2016-01-12

**Authors:** Mélissa Jean, Lajos Gera, Xavier Charest-Morin, François Marceau, Hélène Bachelard

**Affiliations:** ^1^Axe Endocrinologie et Néphrologie, Centre de Recherche, Centre Hospitalier Universitaire de Québec, QuébecQC, Canada; ^2^Department of Biochemistry, University of Colorado Denver, DenverCO, USA; ^3^Axe Maladies Infectieuses et Immunitaires, Centre de recherche, Centre Hospitalier Universitaire de Québec, Université Laval, QuébecQC, Canada

**Keywords:** bradykinin, angiotensin converting enzyme, arginine carboxypeptidases, B_2_ receptors, B-9972, hypotension, blood flow

## Abstract

We reported evidence of bradykinin (BK) regeneration from C-terminal extended BK sequences that behave as peptidase-activated B_2_ receptor (B_2_R) agonists. Further to these *in vitro* studies, we carried out *in vivo* experiments to verify hemodynamic effects of BK analogs exhibiting variable susceptibility toward vascular and blood plasma peptidases. Rats were anesthetized and instrumented to record blood pressure and heart rate responses to bolus intravenous (i.v.) injection of increasing doses of BK, B-9972 (D-Arg-[Hyp^3^,Igl^5^,Oic^7^,Igl^8^]-BK), BK-Arg, BK-His-Leu or BK-Ala-Pro, in the absence or presence of specific inhibitors. In some experiments, pulsed Doppler flow probes measured hindquarter Doppler shift in response to i.v. injections of kinins. BK caused rapid, transient and dose-related hypotensive effects. These effects were potentiated ∼15-fold by the angiotensin converting enzyme (ACE) inhibitor, enalaprilat, but extensively inhibited by icatibant (a B_2_R antagonist) and not influenced by the Arg-carboxypeptidase (CP) inhibitor (Plummer’s inhibitor). The hypotensive responses elicited by the peptidase-resistant B_2_R agonist, B-9972, were not affected by enalaprilat, but were inhibited by icatibant. The hypotensive responses to BK-Arg were abolished by pre-treatment with either the Arg-CP inhibitor or icatibant, pharmacologically evidencing BK regeneration. The hypotensive effects of BK-His-Leu and BK-Ala-Pro, previously reported as ACE-activated substrates, were abolished by icatibant, but not by enalaprilat. *In vivo* regeneration of BK from these two C-terminally extended analogs with no affinity for the B_2_R must follow alternative cleavage rules involving unidentified carboxypeptidase(s) when ACE is blocked. The transient hypotensive responses to BK and three tested analogs coincided with concomitant vasodilation (increased Doppler shift signal). Together, these results provide *in vivo* evidence that interesting hypotensive and vasodilator effects can be extracted from prodrug peptides that behave as peptidase-activated B_2_R agonists.

## Introduction

Kinins, the bradykinin-related peptides, are blood-derived peptide hormones generated by the enzymatic action of proteases called kallikreins on kininogen precursors in response to a variety of physiological and pathological stimuli, including ischemia and tissue injury (Bergaya et al., 2001; Meneton et al., 2001). Bradykinin (BK), a nine amino-acid vasoactive peptide, exerts a large spectrum of actions implicated in many physiological and pathological processes, such as inflammatory reactions, through its ability to cause vasodilation, hyperemia, vascular leakage, and pain sensation (Leeb-Lundberg et al., 2005; Moreau et al., 2005). BK plays also an important role in the regulation of blood pressure, renal, and cardiac functions, via its ability to activate vascular endothelial cells leading to vasodilation, tissue-type plasminogen (t-PA) release, production of nitric oxide (NO) and mobilization of arachidonic acid (Brown et al., 2000; Moreau et al., 2005). The availability of transgenic and knock out animal models for BK-synthesizing or -catabolic enzymes or BK receptors, as well as parallel experiments with pharmacological receptor antagonists in a variety of species, have strengthened the evidence that BK has cardiac and renal protective roles (Yang et al., 1997; Bascands et al., 2003; Griol-Charhbili et al., 2005; Kakoki et al., 2007; Xi et al., 2008). BK exerts its biologic effects by selective activation of two distinct G protein coupled receptors termed BK B_2_ and B_1_ receptors (B_2_R, B_1_R). The B_2_R is constitutively expressed in many tissues, and its activation is believed to play a major role in the cardioprotective effects of BK during hypertension and other clinical and experimental conditions, such as cardiac failure, ischemia, myocardial infarction, and pulmonary hypertension (Heitsch, 2003; Veeravalli and Akula, 2004; Xi et al., 2008; Marketou et al., 2010; Sharma and Al-Banoon, 2012; Potier et al., 2013). Vasodilation and endothelial release of NO and tPA are examples of potentially salutary effects mainly concerning kinins and the endothelial cell B_2_Rs (Pretorius et al., 2003; Leeb-Lundberg et al., 2005). In contrast, the B_1_R appears to have limited distribution and is generally absent in healthy mammalian tissues, but is strongly inducible within few hours under conditions of inflammation and tissue damage (Marceau et al., 1998; Leeb-Lundberg et al., 2005).

BK has a very short half-life (<15 s, Linder et al., 1990) being rapidly inactivated in circulation by non-specific exo- or endopeptidases, commonly referred to as kininases. The kininase I category now includes various arginine-carboxypeptidases (carboxypeptidases N, M, possibly D) that produce des-Arg^9^-BK from BK; this rather minor metabolic pathway is, however, important because it produces the optimal agonists of the B_1_Rs (Cyr et al., 2001). Kininase II, identical to angiotensin I-converting enzyme (ACE), is present in plasma and vascular endothelial cells throughout the body. It inactivates kinins by initially removing the C-terminal dipeptide Phe-Arg from the substrate. ACE inhibitors are widely used in the therapy of cardiovascular and renal diseases (Izzo and Weir, 2011). Although the cardioprotective effects of ACE inhibition have been mostly attributed to inhibition of the formation of the vasopressor agent angiotensin II, growing body of evidence obtained in humans and animal models indicates that a fraction of the therapeutic effects of ACE inhibition is mediated by endogenous kinins, especially at the level of preformed and widely expressed B_2_Rs (Gainer et al., 1998; Squire et al., 2000; Pretorius et al., 2003; Leeb-Lundberg et al., 2005; Marketou et al., 2010).

**FIGURE 1:**
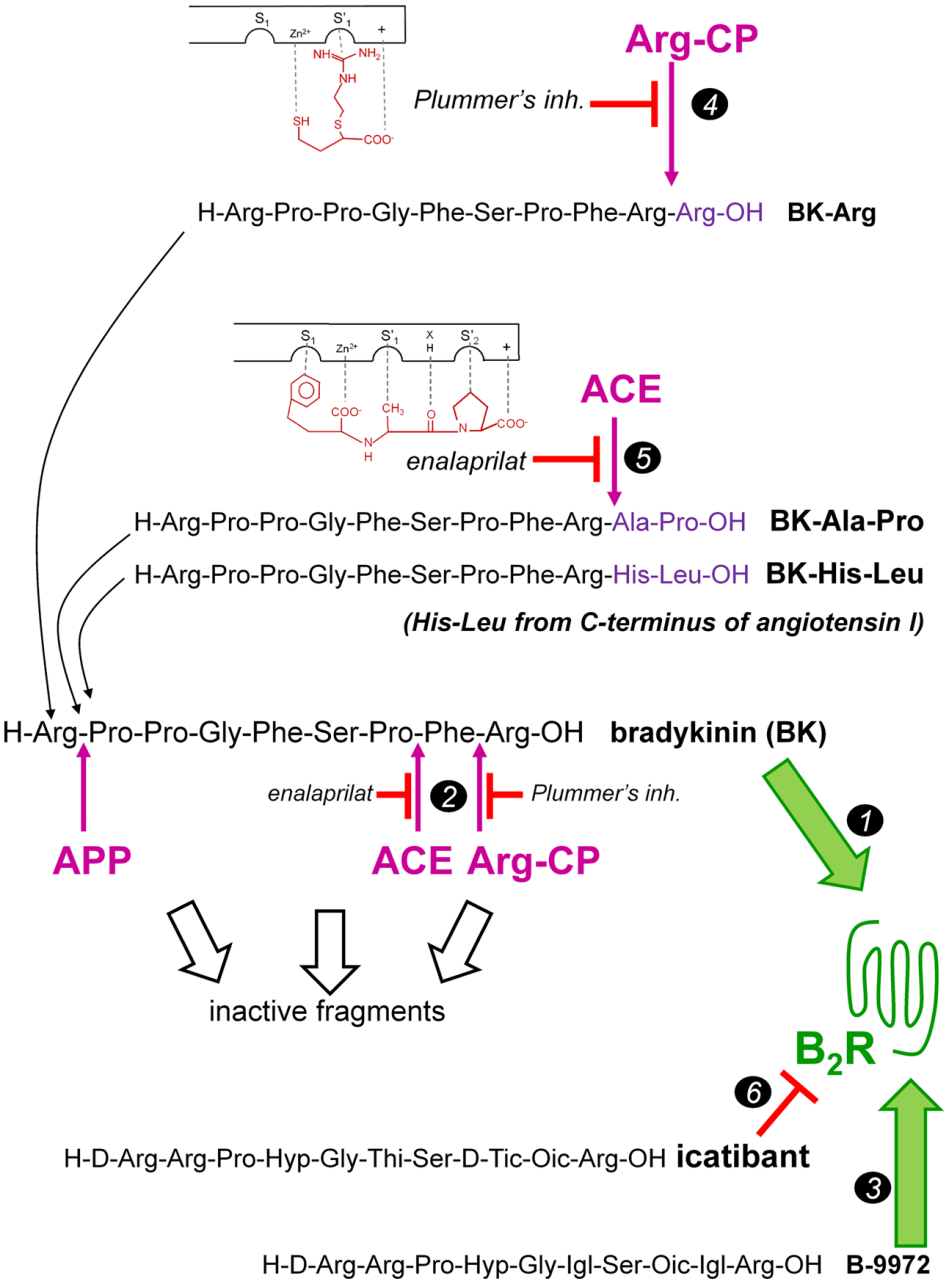
**Previously described BK-related peptides tested *in vivo* as B_2_R agonists in the present study.** BK is a direct high affinity agonist (marker *1*) that is intrinsically fragile, being degraded by several peptidases that terminate its signaling (some mentioned, marker *2*). B-9972 is an inactivation-resistant agonist of the B_2_R by virtue of several of its non-natural residues (marker *3*). Extended BK sequences as potential “prodrug” agonists of the B_2_R activated peptidases were also tested (markers *4*, *5*). Icatibant, a peptide antagonist of the B_2_R, is a further direct ligand of the receptor (marker *6*). The structure of peptidase-specific inhibitors exploited in the present study and their putative binding mode to the active sites of Arg-CP (Plummer’s inhibitor) or ACE (enalaprilat) are indicated (representation modified from Ondetti et al., 1977). Abbreviations: ACE, angiotensin converting enzyme; APP, aminopeptidase P; Arg-CP, arginine carboxypeptidase.

Although there have been very few attempts to use BK or a derivative in pharmacotherapy, the cardiovascular benefits of B_2_R agonists were investigated in rodent models where side effects were not really addressed (Taraseviciene-Stewart et al., 2005; Marketou et al., 2010; Potier et al., 2013). Nevertheless, results were impressive with alleviation of pulmonary hypertension and its cardiac complications (Taraseviciene-Stewart et al., 2005), reduction in acute myocardial damage following ischemia and reperfusion and of fibrotic long term complications of myocardial infarction (Marketou et al., 2010; Potier et al., 2013). All these effects are postulated to stem from BK-induced endothelium-mediated vasodilation, mainly by stimulation of B_2_Rs. Consequently, B_2_R agonists may have important clinical value in the treatment and prevention of various cardiovascular disorders such as hypertension, ischaemic heart disease and other, by mimicking the reported beneficial effects of BK (Heitsch, 2003). Inspired by a “prodrug” strategy where a therapeutic B_2_R agonist would be activated only at the level of vascular endothelial cells, this laboratory recently provided pharmacological evidence of BK regeneration from extended sequences that behave as peptidase-activated B_2_R agonists (Charest-Morin et al., 2014). In continuity with this work, we undertook the present *in vivo* study in healthy rats to assess the feasibility of extracting beneficial vascular effects of stimulation of endothelial B_2_Rs using a variety of ligand design strategies that mainly exploit the susceptibility of these ligands (peptidase-resistant B_2_R agonists and “prodrug” peptides extended around the BK sequence) toward resident vascular peptidases. The BK-related peptides tested and their hypothetical metabolism are presented in **Figure 1.** The natural BK sequence was compared to the peptidase-resistant agonist, B-9972, and to C-terminally prolonged BK homologs designed to retain little affinity for the B_2_R (**Table 1**), but that are presumably activated by vascular or blood plasma peptidases, such as ACE (e.g., BK-His-Leu and BK-Ala-Pro with good affinity for ACE, **Table 1**) or arginine carboxypeptidases (BK-Arg).

**Table 1:** *In vitro* pharmacology of selected B_2_R agonists or BK homologs with C-terminal extensions established in radioligand binding competition assays.

Unlabeled competitor	[^3^H]BK binding to recombinant B_2_R-GFP		[^3^H]enalaprilat binding to recombinant ACE	
		
	Relative potency of competitors	Reference	Relative potency of competitors	Reference
BK	100		100	
B-9972	14.3	Bawolak et al., 2007	0.9^a^	Koumbadinga et al., 2010
BK-Arg	3.47	Charest-Morin et al., 2014	9.6	Present **Figure 5C**
BK-His-Leu	0.3	Charest-Morin et al., 2014	29.5	Charest-Morin et al., 2014
BK-Ala-Pro	0.2	Charest-Morin et al., 2014	44.7	Charest-Morin et al., 2014


*^a^binding to ACE naturally expressed in HUVECs.*

## Materials and Methods

### Experimental Animals and Care

All experimental methods and animal care procedures were reviewed and approved by the Animal Care and Handling Committee of Laval University, in accordance with the Canadian Council on Animal Care. Experiments were performed on male Sprague-Dawley rats (300–375 g) purchased from Charles River Laboratories (St-Constant, QC, Canada). The rats were housed two per cage in a temperature-controlled room (22 ± 1°C) on a 12:12-h light-dark cycle (lights on at 0600). Animals had free access to normal chow diet and tap water. They were allowed to acclimate to their environmental conditions for 1 week prior to being studied.

### [^3^H]Enalaprilat Binding Competition Assay

The affinity of BK and its analogs for ACE has been previously estimated using the displacement of [^3^H]enalaprilat binding to the enzyme expressed in cells (summarized in **Table 1**), except for BK-Arg. To compare the affinity of BK-Arg to that of BK for ACE, HEK 293a cells that transiently expressed human recombinant ACE C-terminally fused with the mCherry fluorescent protein were used to perform a [^3^H]enalaprilat binding assay as described (Koumbadinga et al., 2010) (the ACE variant construction retains its high affinity for the radioligand; Charest-Morin and Marceau, in preparation). A 2 nM concentration of [^3^H]enalaprilat was used to generate binding competition curves.

### Surgical Preparation

At the end of the acclimation period, the rats were anesthetized with sodium pentobarbital (50 mg kg^-1^, i.p., supplemented as required) and had two separate catheters implanted; one into the right jugular vein [for intravenous (i.v.) injection] and the other into the left femoral artery (for direct measurement of blood pressure and heart rate (HR). In some experiments, a pulsed Doppler flow probe (Haywood et al., 1981) was also implanted around the distal abdominal aorta (below the ileocecal artery) through a midline abdominal incision to monitor changes in hindquarter hemodynamic, according to the method developed by Gardiner and Bennett (1988) and as previously described (Bachelard et al., 1992b). The probes wires were connected to a pulsed Doppler monitoring system (VF-1 Doppler flowmeter; Crystal Biotech, Holliston, MA, USA) modified to operate with a pulse repetition frequency of 125 kHz (Gardiner et al., 1990) and fitted with HVPD-20 modules. The mean Doppler signal represents the average hindquarter blood flow velocity, a relative index of blood flow. The accuracy of the pulsed Doppler method in detecting changes in regional blood flow and vascular resistance was established by the demonstration of a significant correlation between velocity recorded from the Doppler unit and volume flow recorded simultaneously (Haywood et al., 1981). Experiments started at least 30 min following the end of surgery in anesthetized rats.

### Blood Pressure and Heart Rate Measurements

The femoral arterial catheter implanted to monitor direct blood pressure was connected to a pressure transducer (TSD 104A, Biopac Systems Inc., Goleta, CA, USA) coupled to a computer-based data acquisition system (MP100, Biopac Systems Inc., Goleta, CA, USA) to continuously record pulsate arterial pressure. The animals were maintained under anesthesia and were warmed with a heated pad. The mean arterial pressure and HR were simultaneously calculated by the Acknowledge software (version 3.9.1) for Windows (Biopac Systems Inc., Goleta, CA, USA) and displayed continuously.

### *In Vivo* Vascular Reactivity

The experiments were initiated after allowing at least 20 min for stabilization. Then, baseline measurements of HR and phasic and mean arterial blood pressure (MAP) were made over a period of 10 min. A dose response curve was then obtained by recording changes in blood pressure and HR elicited by i.v. injection of peptide vehicle followed by increasing doses (0.025, 0.1, 0.4, 1.6 et 6.4 μg/kg) of one of these peptides: BK, BK-His-Leu, B-9972, BK-Arg, BK-Ala-Pro or des-Arg^9^-BK. Peptides were dissolved in isotonic saline (0.9% NaCl) containing 0.1% BSA to prevent the adsorption of peptide to the glassware and plastic surfaces. All i.v. injections were given as 100 μl boluses which were washed in with a further 100 μl of saline (the dead space of the catheter). Only one peptide was tested per group of rats and each injection started with saline-BSA 0.1% followed by the lowest dose of peptide. The next dose was administered once all recorded cardiovascular parameters had returned to baseline after the previous injection (usually 2–10 min). At the end of the experiments each animal was euthanized with an overdose of sodium pentobarbitone (240 mg/kg, i.v.).

The mechanism subserving the cardiovascular responses to i.v. injections of increasing doses of the different BK agonists was first investigated in animals pretreated with the ACE inhibitor, enalaprilat. In these experiments, the animals were separated in five groups depending on the agonist tested. Enalaprilat was intravenously administered as bolus (0.1 mg/kg, 0.1 ml) following a 10 min period of baseline measurements of HR and blood pressure. Fifteen minutes later, dose-response curves to BK, BK-His-Leu, B-9972, BK-Arg, or BK-Ala-Pro were obtained in the indicated group of rats, as described above. Further experiments were made in rats pretreated with the Plummer’s inhibitor (mercaptomethyl- 3-guanidinoethylthiopropanoic acid), a high affinity inhibitor of arginine carboxypeptidases that is an arginine analog (Plummer and Ryan, 1981). In these experiments, the inhibitor was intravenously administered as bolus (0.75 mg/kg, 0.1 ml) followed 15 min later by the i.v. injection of increasing doses of BK or BK-Arg, in two different groups of rats. Further dose-response curves were also obtained from rats pretreated with a potent, long acting and selective B_2_R antagonist, icatibant (Hoe 140) (D-Arg-[Hyp^3^, Thi^5^, D-Tic^7^, Oic^8^] bradykinin) (Hock et al., 1991; Wirth et al., 1991; Rhaleb et al., 1992; Marceau et al., 1994). In these experiments, icatibant was intravenously administered as bolus (10 μg/kg, 0.1 ml) 15 min before the i.v. injection of increasing doses of BK, BK-His-Leu, B-9972, BK-Ala-Pro, or BK-Arg, in separated groups of rats, as above. The doses of different inhibitors were based on preliminary experiments and from studies performed by others (Ishida et al., 1989; Wirth et al., 1991; Muto et al., 2003), with deliberate low dosing for peptidase inhibitors for the sake of their selectivity of action. Whether a component of BK hemodynamic effects is mediated by the B_1_R was assessed in rats pre-treated with the specific B_1_R antagonist, B-9858 (20 μg/kg, 0.1 ml). B-9858 (Lys-Lys-[Hyp^3^, Igl^5^, D-Igl^7^, Oic^8^]des-Arg^9^-BK) is a selective B_1_R antagonist (Leeb-Lundberg et al., 2005) that has been found active at the rat B_1_R (Scott et al., 2003).

### Acute Hindquarter Hemodynamic Effects in Anesthetized Rats

Before each experiment, a 30-min stabilization period was allowed, during which continuous recording of instantaneous HR, phasic, and MAP and phasic and mean Doppler shift signals from the hindquarter probe were made in anesthetized rats, using the Biopac data acquisition and analysis system described above. The purpose of recording phasic Doppler shift signals was to ensure that they were of acceptable quality during the experiments. Then, the rats received i.v. injections of peptide vehicle (saline-BSA 0.1%), BK (700 ng/kg), B-9972 (150 ng/kg), BK-Arg (2 μg/kg), and BK-His-Leu (400 ng/kg) in random order. For each agent, the dose interpolated from the dose-response curves produces a sizeable hypotensive effect of 15–30 mmHg. All i.v. injections were given as 100 μl boluses which were washed in with a further 100 μl of saline. The next dose was administered once all recorded cardiovascular parameters had returned to baseline after the previous injection (usually 5–7 min). At the end of the experiments the rats were euthanized with an overdose of anesthetic (pentobarbital 240 mg/kg, i.v.).

### Drugs

BK and des-Arg^9^-BK were purchased from Bachem (Torrance, CA, USA), the B_2_R antagonist icatibant, from Phoenix Pharmaceuticals (Burlingame, CA, USA), enalaprilat dehydrate, from Kemprotec Ltd. (Maltby, Middlesbrough, UK) and the Plummer’s carboxypeptidase inhibitor, from Calbiochem (La Jolla, CA, USA). B-9972 (D-Arg-[Hyp^3^,Igl^5^,Oic^7^,Igl^8^]-BK) is a peptidase-resistant agonist of the B_2_R (Bawolak et al., 2007, 2009) that was recently resynthesized and purified by one of us (L.G.). B-9858 has also been designed, produced and characterized by us (Larrivée et al., 2000). C-terminally extended BK sequences (BK-Arg, BK-His-Leu, BK-Ala-Pro) were custom synthesized and characterized by Peptide 2.0 Inc. (Chantilly, VA, USA) as described (Charest-Morin et al., 2014).

### Statistical Analysis

Data are presented as means ± SEM. Radioligand binding data were fitted by non-linear regression to a one-site competition equation using a least-square method (Prism 4.0, GraphPad Software Inc., San Diego, CA, USA) and IC_50_ values calculated from this procedure. Data describing baseline values of HR and MAP and hypotensive responses to peptides in anesthetized rats were assessed by using one-way analysis of variance (ANOVA) followed by the Dunnett’s test (repeated comparison with a common control). The effects of peptidase inhibitors on the hypotensive responses to BK and alternate kinin receptor agonists were assessed by using ANOVA followed by the Dunnett’s test. A value of *P* < 0.05 was considered significant.

## Results

### Dose Response Effects of BK on Mean Arterial Blood Pressure and HR

Baseline values for MAP and HR measured in the untreated control group or 15 min after i.v. pretreatment with enalaprilat, icatibant or the Plummer’s inhibitor are shown in **Table 2.** While no significant changes in basal values of MAP were noted between the enalaprilat and Plummer’s inhibitor pretreated groups, a slight but significant reduction in basal MAP was found between the icatibant pretreated group and the control group. Furthermore, slight but significant reductions in basal HR were noted between the icatibant and Plummer’s inhibitor pretreated groups and the control group, while a small significant increase in basal HR was found between the enalaprilat pretreated groups and the control group.

**Table 2:** Basal cardiovascular parameters in anesthetized, pre-treated rats.

Pre-treatment	Mean arterial blood pressure (MAP)	Heart rate (HR)	*n*
Control	103.1 ± 1.8	393 ± 6	47
Enalaprilat	99.2 ± 1.5	416 ± 6ˆ*	39
Plummer’s inhibitor	107.1 ± 4.7	353 ± 17ˆ*	12
Icatibant	95.3 ± 1.6ˆ*	356 ± 8ˆ**	25
B-9858	103.3 ± 6.2	397 ± 11	7
ANOVA	*F* = 3.06, *P* = 0.011	*F* = 10.69, *P* < 10^-4^	


*^∗^*P* < 0.05; ^∗∗^*P* < 0.01, Dunnett’s test vs. Control.*

**FIGURE 2:**
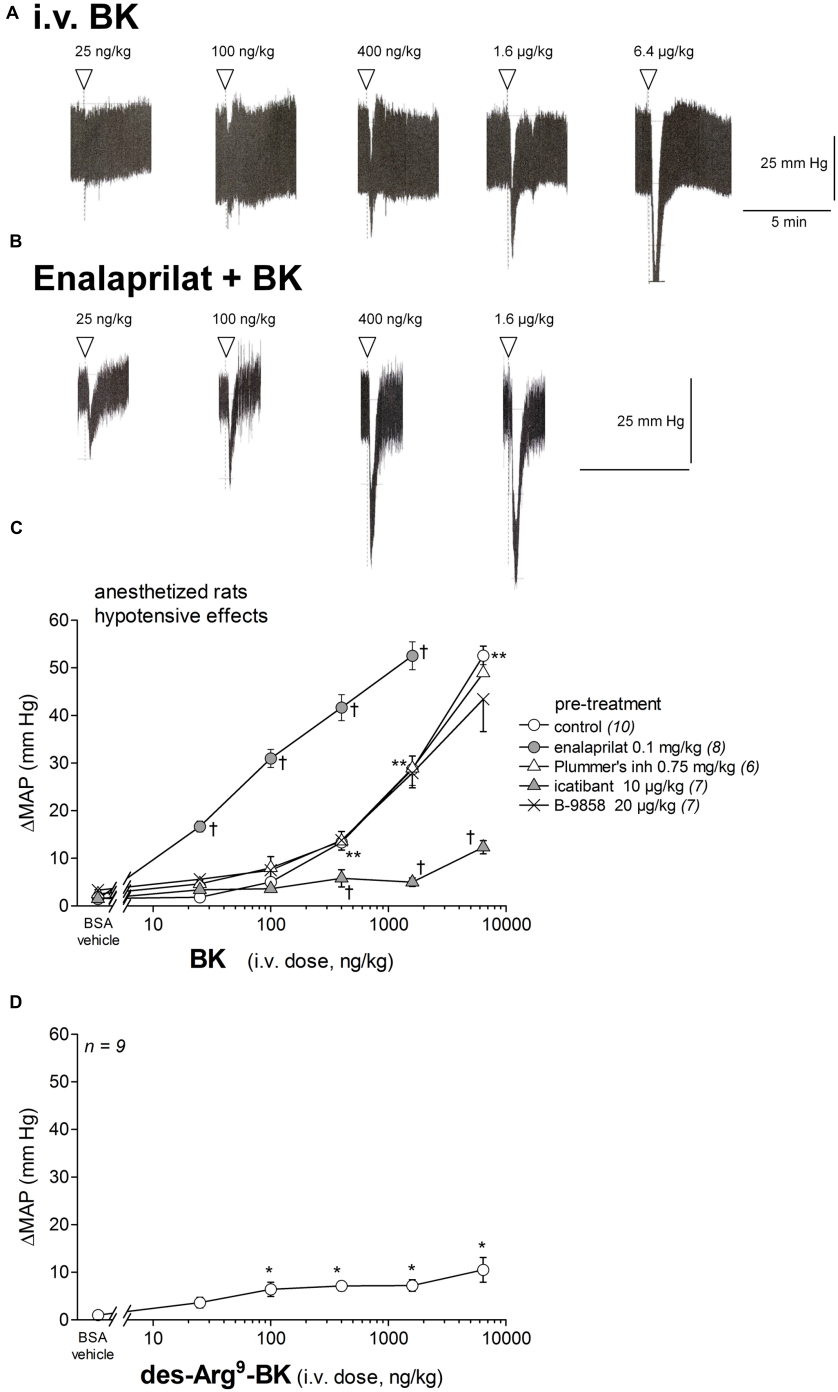
**Hypotensive responses to i.v. bolus injections of increasing doses of bradykinin (BK) and des-Arg^9^-BK in anesthetized rats.** **(A,B)** Representative traces showing the dose-response effect of BK on systemic blood pressure in untreated rats **(A)** and in rats pretreated with enalaprilat **(B)**. Enalaprilat (0.1 mg/kg) was i.v given as bolus 15 min before starting the injections of BK. Doses of BK are in ng/kg. Abscissa: time; ordinate: arterial pressure (mm Hg). **(C)** Effect of i.v. pretreatment with enalaprilat (0.1 mg/kg), icatibant (10 μg/kg), Plummer’s inhibitor (0.75 mg/kg) or B-9858 (20 μg/kg) on the maximal hypotensive response to i.v. injections of increasing doses of BK. The pretreatment with each peptidase inhibitor was given as bolus 15 min before starting the injections of BK. The number of determination in different animals is given between parentheses for control and each inhibitor. **(D)** Dose-effect for the maximal hypotensive response to des-Arg^9^-BK (*n* = 9). Abscissa: dose (ng/kg); ordinate: fall of mean arterial blood pressure (MAP; mm Hg). Values are means ± SEM, shown by vertical lines. *^∗^P* < 0.05; ^∗∗^*P* < 0.01 control group values compared with vehicle value; ^†^*P* < 0.01 pretreated group values compared with the control group values (Dunnett’s test).

**FIGURE 3:**
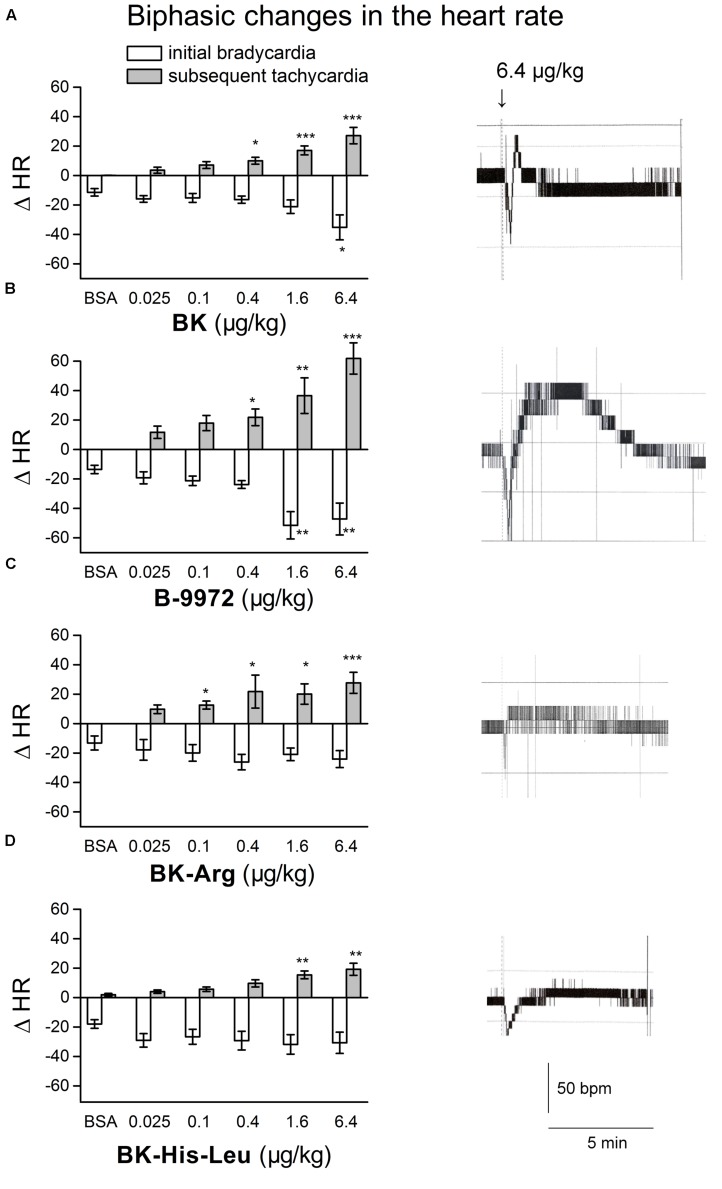
**Bar graph showing maximal changes in heart rate (HR) elicited by i.v. injections of vehicle, and increasing doses of **(A)** BK (*n* = 10), **(B)** B-9972 (*n* = 7), **(C)** BK-Arg (*n* = 11), and **(D)** BK-His-Leu (*n* = 10) in anesthetized rats.** On the right side of each graph are presented typical tracings showing the changes in HR elicited by the highest dose of the agonist tested. Abscissa: dose (μg/kg); ordinate: rises of HR (bpm). Values are means ± SEM. ^∗^*P* < 0.05; ^∗∗^*P* < 0.01; ^∗∗∗^*P* < 0.001 compared with vehicle value (Dunnett’s test).

As expected and shown in **Figures 2A,B**, the i.v. injection of increasing doses of BK in anesthetized rats caused a rapid and transient dose-related decrease in MAP; responses were significantly greater than the saline-BSA vehicle for BK doses superior or equal to 0.4 μg/kg (*P* < 0.01, Dunnett’s test). Tachycardia, (preceded by a significant bradycardia only at the dose of 6.4 μg/kg), accompanied the hypotensive responses only at 0.4 μg/kg and above (**Figure 3**). In rats pretreated with enalaprilat, the hypotensive effect of BK was potentiated ∼15-fold (**Figure 2C**); the duration of the hypotensive episodes were not different from those of controls (**Figures 2A,B**). Pretreatment with icatibant significantly and extensively inhibited the dose-dependent hypotensive response to BK in the 0.4–6.4 μg/kg dose range (*P* < 0.01, Dunnett’s test; **Figure 2C**), while pretreatment with the Plummer’s inhibitor had no effect (**Figure 2C**), consistent with a minor role of Arg-carboxypeptidases in the metabolism of BK. The B_1_R agonist des-Arg^9^-BK had no significant effect on MAP at the dose of 0.025 μg/kg, while slight but significant decreases in MAP were noted at 0.1 μg/kg and above (*P* < 0.05, Dunnett’s test) when compared to vehicle values (**Figure 2D**). The comparatively small effects of the fragment des-Arg^9^-BK exclude that responses to BK are mediated by the B_1_Rs via metabolism by Arg-carboxypeptidases. Accordingly, the B_1_R antagonist B-9858, did not significantly modify the effect of BK (**Figure 2C**).

### Dose Response Effects of Alternate Kinin Receptor Agonists on MAP

**FIGURE 4:**
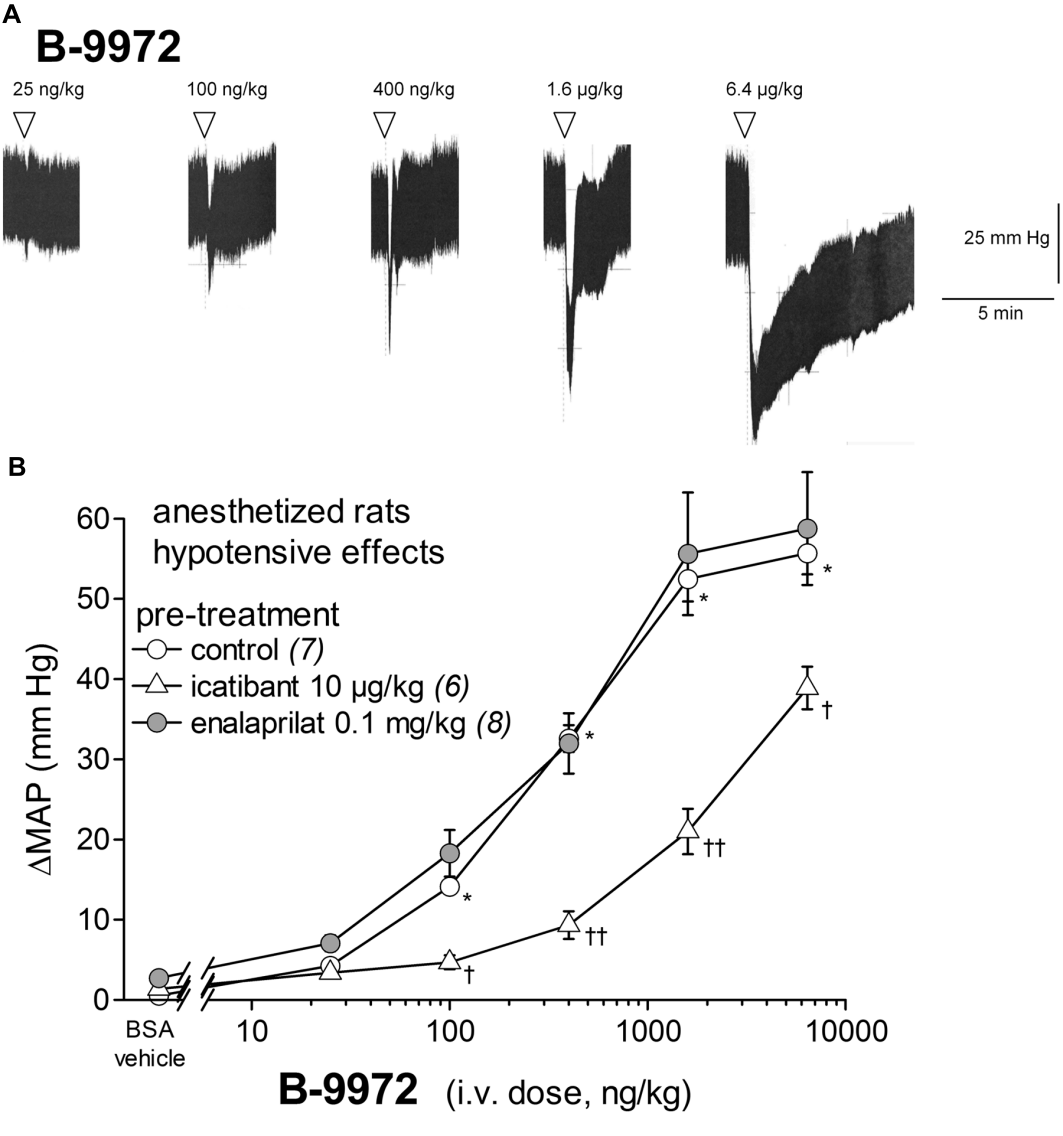
**Hypotensive responses to i.v. bolus injections of increasing doses of B-9972 in anesthetized rats.** **(A)** Representative traces showing the dose-response effect of B-9972 on systemic blood pressure. Doses of B-9972 are in ng/kg. Abscissa: time; ordinate: arterial pressure (mm Hg). **(B)** Effect of i.v. pretreatment with enalaprilat (0.1 mg/kg) or icatibant (10 μg/kg) on the maximal hypotensive response to i.v. injections of increasing doses of B-9972. The pretreatment with the peptidase inhibitors was given as bolus 15 min before starting the injections of B-9972. The number of determination in different animals is given between parentheses for control and each inhibitor. Abscissa: dose (ng/kg); ordinate: fall of (MAP; mm Hg). Values are means ± SEM shown by vertical lines. ^∗^*P* < 0.01 control group values compared with vehicle value; ^†^*P* < 0.05; ^††^*P* < 0.01 pretreated group values compared with the control group values (Dunnett’s test).

The i.v. injection of increasing doses of the peptidase-resistant B_2_R agonist B-9972 decreased MAP in a dose-dependent manner (**Figures 4A,B**). The hypotensive response elicited by B-9972 was significant at doses of 0.1 to 6.4 μg/kg when compared to vehicle values (*p* < 0.01, Dunnett’s test). The hypotensive episodes induced by B-9972 had the same temporal profile than those elicited by BK (**Figures 4A** and **2A**) except for the highest dose of the analog, that was associated with a half-recovery time significantly longer than that of a similar dose of BK (*P* < 0.01, Dunnett’s test). Consistent with its low affinity for ACE (**Table 1**) and constrained C-terminal structure, the dose-dependent hypotensive response to B-9972 was not affected by pretreatment with enalaprilat. However, consistent with its direct agonist action on the B_2_R, the effects of the 0.1–1.6 μg/kg doses of B-9972 were significantly abated by pretreatment with icatibant; thus this competitive antagonist reduced the apparent potency of B-9972 by about 10-fold (**Figure 4B**). Biphasic effects of B-9972 on the HR were generally comparable to that of BK but of higher amplitudes at 1.6–6.4 μg/kg (**Figure 3**).

**FIGURE 5:**
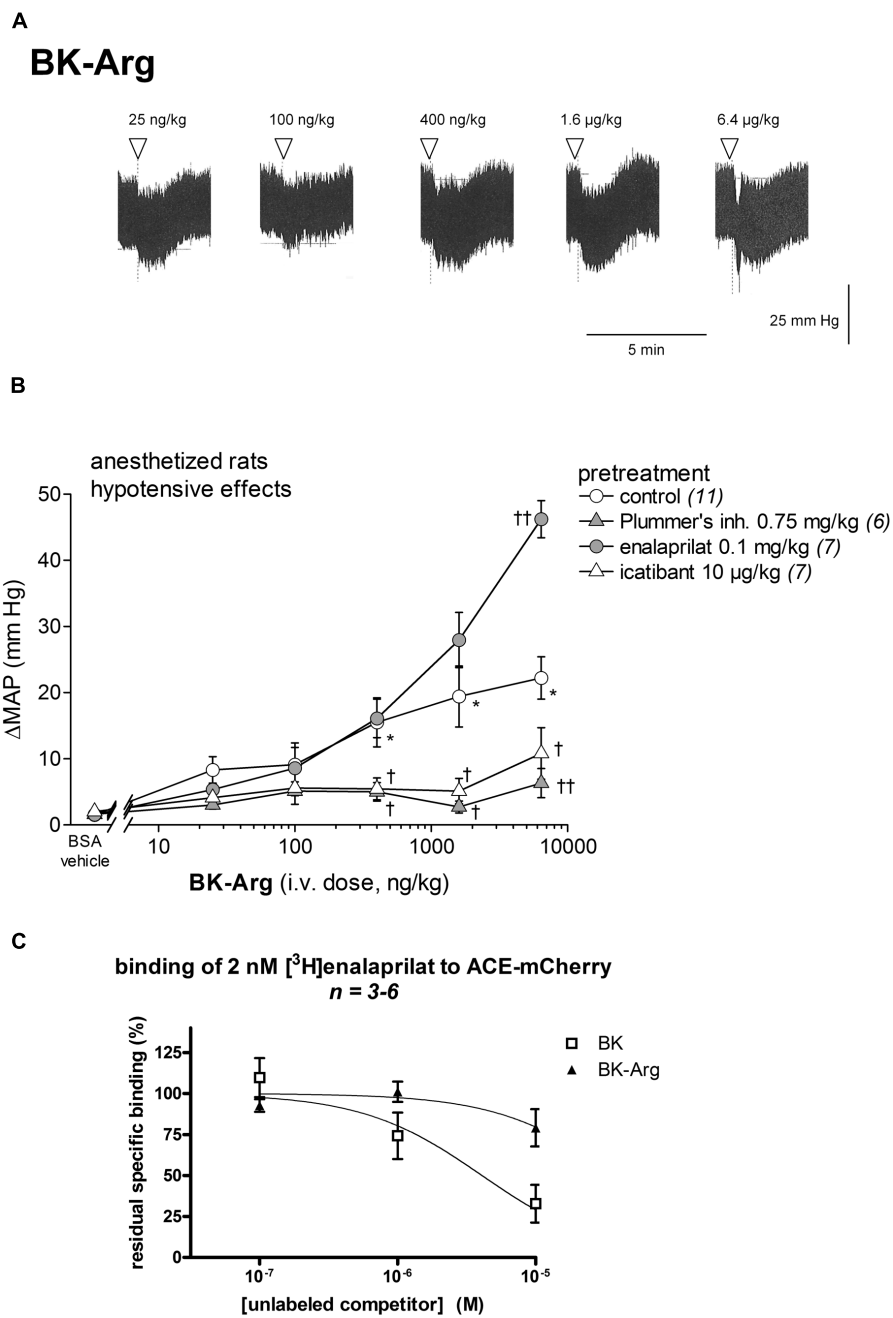
**Hypotensive responses to i.v. bolus injections of increasing doses of BK-Arg in anesthetized rats.** **(A)** Representative traces showing the dose-response effect of BK-Arg on systemic blood pressure. Doses of BK-Arg are in ng/kg. Abscissa: time; ordinate: arterial pressure (mm Hg). **(B)** Effect of i.v. pretreatment with enalaprilat (0.1 mg/kg), icatibant (10 μg/kg) or Plummer’s inhibitor (0.75 mg/kg) on the maximal hypotensive response to i.v. injections of increasing doses of BK-Arg. The pretreatment with the peptidase inhibitors was given as bolus 15 min before the starting injections of BK-Arg. The number of determination in different animals is given between parentheses for control and each inhibitor. Abscissa: dose (ng/kg); ordinate: fall of (MAP; mm Hg). Values are means ± SEM. **(C)** Competition of [^3^H]enalaprilat (2 nM) binding to recombinant human somatic ACE by BK-Arg and BK. Results are expressed as the residual specific binding (means ± SEM of n duplicate determinations). ^∗^*P* < 0.01 control group values compared with vehicle value;^†^*P* < 0.05; ^††^*P* < 0.01 pretreated group values compared with the control group values (Dunnett’s test).

As shown in **Figures 5A,B**, the i.v. injection of increasing doses of BK-Arg elicited significant and dose-dependent decreases in MAP. The reductions in MAP were significant at doses of 0.4 to 6.4 μg/kg (*P* < 0.01, Dunnett’s test) when compared to vehicle values. This peptide is predicted to be an indirect activator of the B_2_R, via its conversion to BK (**Figure 1**; **Table 1**). Consistently, its *in vivo* effects were abolished in rats pre-treated with either the Plummer’s carboxypeptidase inhibitor or icatibant (**Figure 4B**; effects of BK-Arg 0.4–6.4 μg/kg significantly inferior in pretreated animals, *P* < 0.05, Dunnett’s test). BK regeneration from BK-Arg should not involve the carboxydipeptidase ACE (Charest-Morin et al., 2014). To verify that ACE does not have a high affinity for BK-Arg, we performed a competition assay involving [^3^H]enalaprilat binding to recombinant ACE (**Figure 5C**). While micromolar concentration levels of BK displace the radioligand from the peptidase, as previously reported, BK-Arg at 10 μM had little effect in this competition assay (**Figure 5C**; extrapolated relative potency of 9.6% compared with that of BK, **Table 1**). Whereas pretreatment with enalaprilat did not affect the hypotensive response to 0.025 to 1.6 μg/kg BK-Arg, the pretreatment was found to significantly potentiate the hypotensive response elicited by the highest dose of BK-Arg (6.4 μg/kg; *P* < 0.01, Dunnett’s test). This might result from the potentiation of regenerated BK, considering that BK-Arg has a low intrinsic affinity for ACE (**Table 1**; **Figure 5C**). Tachycardia accompanied the hypotensive responses at doses of 0.1 to 6.4 μg/kg (*P* < 0.01, Dunnett’s test, **Figure 3**).

**FIGURE 6:**
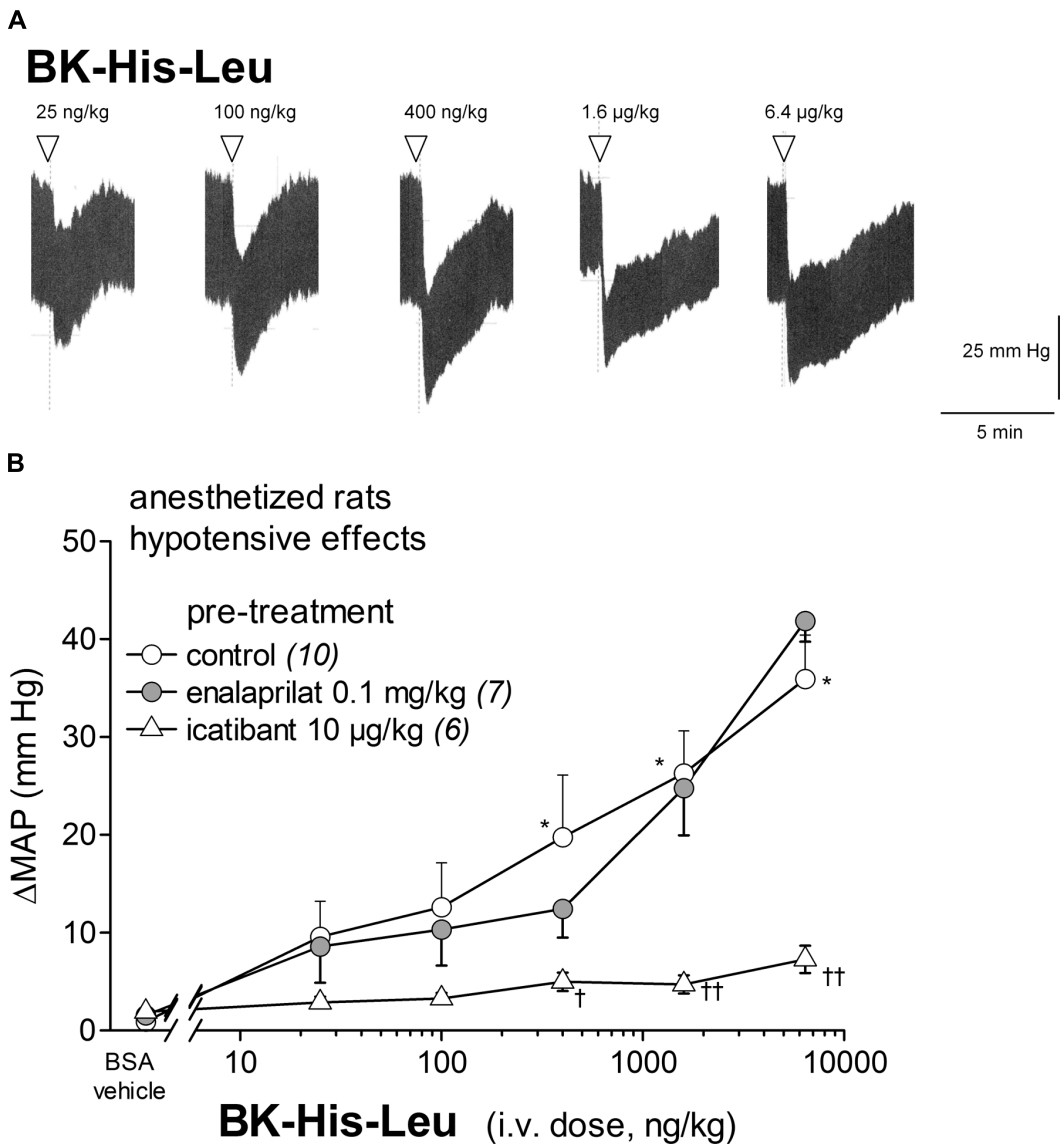
**Hypotensive responses to i.v. bolus injections of increasing doses of BK-His-Leu in anesthetized rats.** **(A)** Representative traces showing the dose-response effect of BK-His-Leu on systemic pressure. Doses of BK-His-Leu are in ng/kg. Abscissa: time; ordinate: arterial pressure (mm Hg). **(B)** Effect of i.v. pretreatment with enalaprilat (0.1 mg/kg) or icatibant (10 μg/kg) on the maximal hypotensive response to i.v. injections of increasing doses of BK-His-Leu. The pretreatment with the peptidase inhibitors was given as bolus 15 min before starting the injections of BK-His-Leu. The number of determination in different animals is given between parentheses for control and each inhibitor. Abscissa: dose (ng/kg); ordinate: fall of (MAP; mm Hg). Values are means ± SEM. ^∗^*P* < 0.01 control group values compared with vehicle value; ^†^*P* < 0.05; ^††^*P* < 0.01 pretreated group values compared with the control group values (Dunnett’s test).

**Figures 6A,B** shows that BK-His-Leu (0.025–6.4 μg/kg) decreased MAP in a significant and dose-dependent manner. The reductions in MAP were significant at doses of 0.4 to 6.4 μg/kg (*P* < 0.01, Dunnett’s test) when compared to vehicle values. BK-His-Leu is predicted to be an indirect activator of the B_2_R, via its conversion to BK, which should involve the carboxydipeptidase ACE (**Figure 1**). Surprisingly, the dose-dependent hypotensive response to BK-His-Leu, which has very little affinity for the B_2_R but a good one for ACE (**Table 1**), was not significantly affected by pretreatment with enalaprilat. However, the hypotensive effect of BK-His-Leu was abolished by pretreatment with icatibant (effects significantly inferior to those of control animals in the 0.4–6.4 μg/kg dose range, Dunnett’s test, **Figure 6B**), raising the possibility that in the presence of enalaprilat, BK regeneration from BK-His-Leu might involve unidentified peptidase(s) via alternate cleavage rules. Tachycardia accompanied the hypotensive responses at doses of 1.6 and 6.4 μg/kg (*P* < 0.01, Dunnett’s test, **Figure 3**).

Both pro-drug peptides BK-Arg and BK-His-Leu had a tendency to produce prolonged hypotensive episodes with, occasionally, a slow onset (**Figures 5A** and **6A**); however, this was variable and statistics on recovery half-time for hypotensive episodes generally failed to show statistical difference from those recorded following similar doses of BK.

**FIGURE 7:**
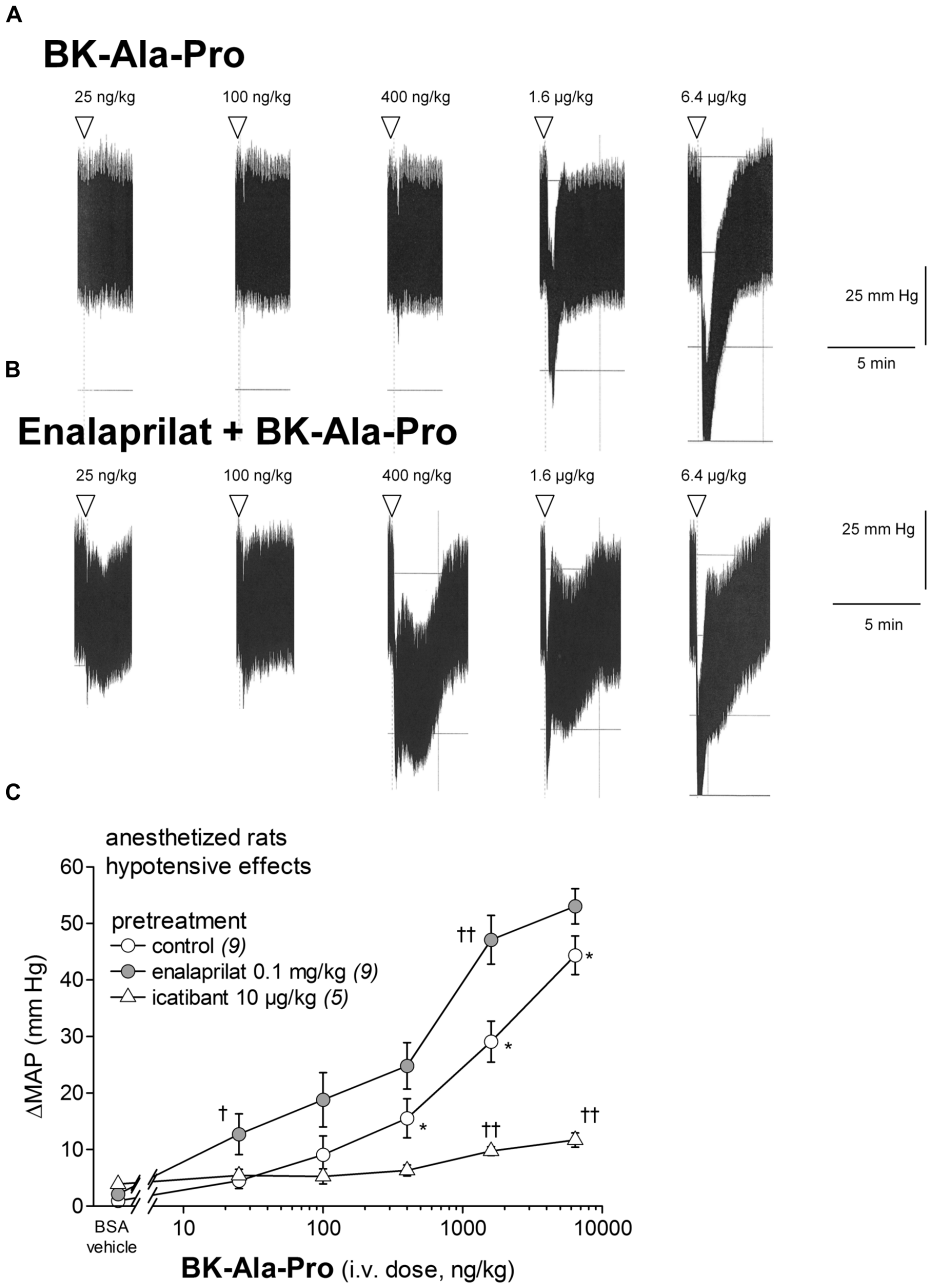
**Hypotensive responses to i.v. bolus injections of increasing doses of BK-Ala-Pro in anesthetized rats.** **(A,B)** Representative traces showing the dose-response effect of BK-Ala-Pro on systemic blood pressure in untreated rats **(A)** and in rats pretreated with enalaprilat **(B)**. Enalaprilat (0.1 mg/kg) was i.v given as bolus 15 min before starting the injections of BK-Ala-Pro. Doses of BK-Ala-Pro are in ng/kg. Abscissa: time; ordinate: arterial pressure (mm Hg). **(C)** Effect of i.v. pretreatment with enalaprilat (0.1 mg/kg) or icatibant (10 μg/kg) on the maximal hypotensive response to i.v. injections of increasing doses of BK-Ala-Pro. The pretreatment with the peptidase inhibitors was given as bolus 15 min before starting the injections of BK-Ala-Pro. The number of determination in different animals is given between parentheses for control and each inhibitor. Abscissa: dose (ng/kg); ordinate: fall of (MAP; mm Hg). Values are means ± SEM. ^∗^*P* < 0.01 control group values compared with vehicle value; ^†^*P* < 0.05; ^††^*P* < 0.01 pretreated group values compared with the control group values (Dunnett’s test).

BK-Ala-Pro is an alternate ACE substrate that may regenerate BK following the action of ACE (**Figure 1**; **Table 1**). **Figure 7** shows that BK-Ala-Pro decreased MAP in a dose-dependent manner (effects different from the BSA-saline vehicle in the 0.4–6.4 μg/kg dose range, *P* < 0.01 for each of these doses). While icatibant abated the effects of BK-Ala-Pro (*P* < 0.01 for the kinin doses of 1.6 and 6.4 μM, Dunnett’s test), enalaprilat pretreatment unexpectedly potentiated this peptide (significantly for doses of 0.025 and 1.6 μg/kg; *P* < 0.05 and 0.01, respectively; **Figure 7C**).

### Hemodynamic Effects of Intravenous Injection of BK and Alternate Kinin Receptor Agonists in Rats

**FIGURE 8:**
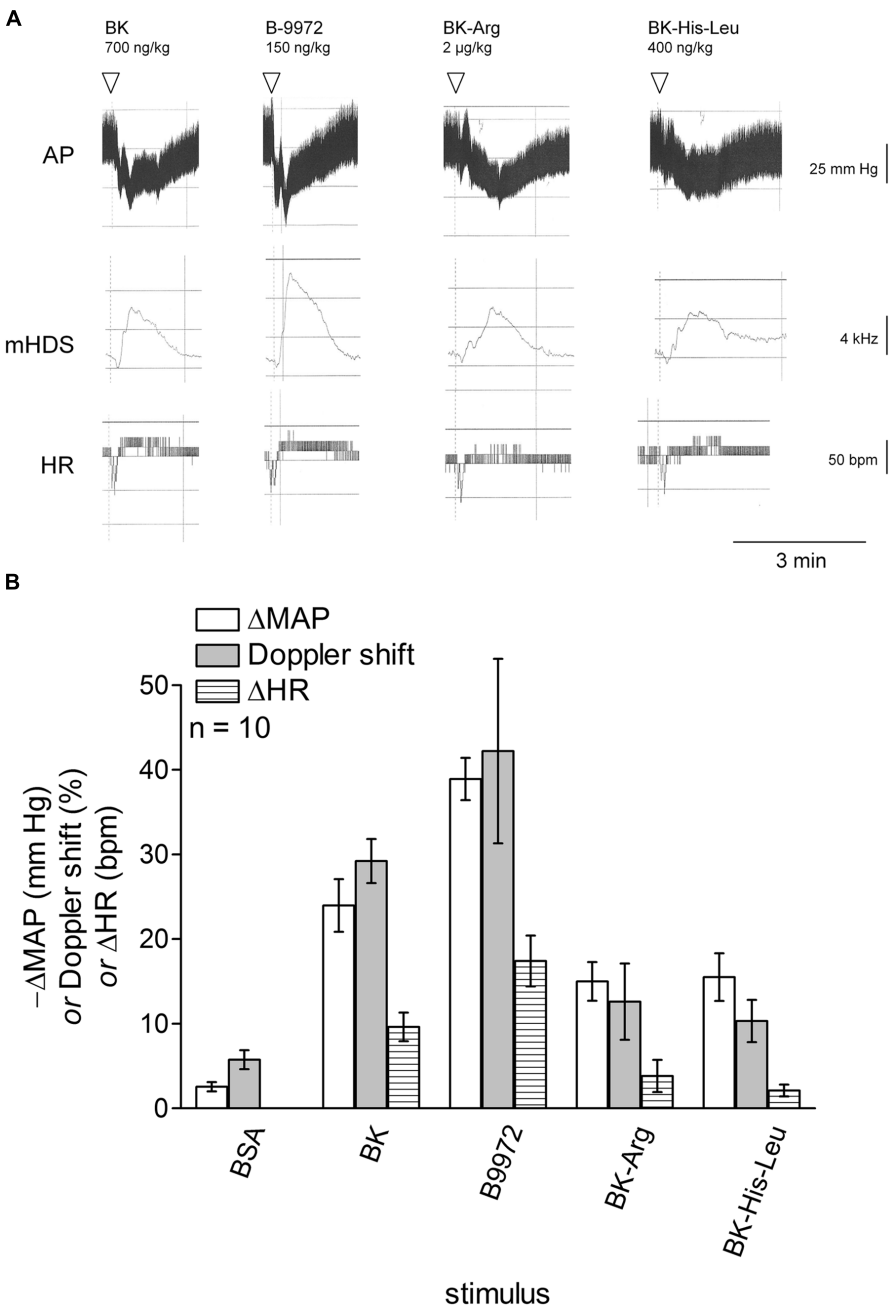
**Cardiovascular responses to i.v. bolus injections of BK and three alternate kinin receptor agonists in anesthetized rats.** **(A)** Simultaneous changes in arterial pressure (AP), mean hindquarter Doppler shift (mHDS) signal and HR elicited by i.v. injections of BK, B-9972, BK-Arg, and BK-His-Leu. Abscissa: time; ordinate: AP (mm Hg), mHDS (kHz); HR (beats per minute, bpm). **(B)** Bar graph showing maximal fall in MAP and rises in mHDS and HR elicited by i.v. injections of vehicle, BK and the three alternate kinin receptor agonists. Abscissa: dose (ng/kg); ordinate: fall of MAP (mm Hg) and rises of mHDS (%) and HR (bpm). Values are means ± SEM. BK (700 ng/kg), B-9972 (150 ng/kg), BK-Arg (2 μg/kg), and BK-His-Leu (400 ng/kg) were randomly administered as bolus in 10 anesthetized rats (see Materials and Methods). See Results for analysis.

**Figures 8A,B** shows the simultaneous changes in phasic and MAP, mean hindquarter Doppler shift (mHDS) signal and HR induced by intravenous injections of vehicle, BK and alternate kinin receptor agonists administered at doses producing a sizeable hypotensive effect of 15–30 mmHg. Thus, we found that, for each tested agonist, the observed hypotensive response coincided with a concomitant increase in the mean Doppler shift signal and tachycardia when compared with vehicle values. The increase in mHDS might reflect a change in vascular resistance (vasodilation) redistributing flow to the hindlimb muscles, as previously shown in conscious rats (Bachelard et al., 1992a). The amplitude of the hypotensive episodes (ΔMAP) predicted the maximal tachycardia and Doppler shift irrespective of the peptide identity (*r* = 0.972 and 0.962, respectively, *P* < 0.01 for each comparison), suggesting actions mediated along a continuum of physiological effects mediated by cardiovascular B_2_Rs.

## Discussion

Aberrant protease signaling pathways have been implicated in several diseases, ranging from cardiovascular disorders to cancer, making them attractive therapeutic targets in drug development. To this end, the development of protease resistant drugs might be very useful in order to prolong beneficial effects of endogenous substances otherwise too rapidly degraded. Protease-activated prodrugs can also be successfully exploited to improve drug delivery to areas where protease expression is higher than in normal tissues, and might contribute to reduce off-target side effects. Although the protective role of kinins in the circulation is increasingly recognized, there have been very few attempts to use BK or a derivative in cardiovascular therapeutics. In the present study, we exploited the distribution of ectopeptidases expressed in the vasculature and blood plasma to examine in anesthetized rats the feasibility of extracting hemodynamic effects of the stimulation of endothelial B_2_Rs using a variety of ligand design strategies that mainly exploit their susceptibility toward vascular peptidases. The vascular and plasmatic localization of ACE and kininase I–type carboxypeptidases makes them ideally situated to regulate BK activity on B_2_Rs of vascular tissue, thus avoiding extravascular stimulation of these receptors (e.g., in sensory nerve terminal, epithelia).

BK is a direct and high affinity B_2_R agonist that is also an effective ACE substrate (**Table 1**). As expected, the rapid and transient hypotensive response to systemic administration of BK was greatly enhanced by pretreatment with an ACE inhibitor, but extensively inhibited in the presence of a B_2_R antagonist, and remained unchanged in the presence of a specific inhibitor of arginine carboxypeptidases (**Figure 2**). These results further confirm the important role played by the ACE, as the main BK-inactivating peptidase in the extracellular space (Cyr et al., 2001). These findings are also consistent with previous claims of a role of BK in the cardiovascular and therapeutic effects of ACE inhibitors, especially at the level of preformed and widely expressed B_2_Rs (Leeb-Lundberg et al., 2005).

We then tested the effects of the stable B_2_R agonist B-9972. This analog integrates several substitutions that make it resistant to extracellular and endosomal inactivation by multiple peptidases/proteases (Bawolak et al., 2007, 2009). It is worth mentioning that, in a rare attempt to evaluate therapeutic actions of BK receptor agonists, it was shown that 4 weeks treatment with the stable agonist B-9972, in a rat model of severe pulmonary hypertension, causes reduction of pulmonary artery pressure and right ventricular hypertrophy, via the classical vasodilator effect mediated by endothelial B_2_Rs (Taraseviciene-Stewart et al., 2005). Here we found that, consistent with its low affinity for ACE and its direct agonist action on the B_2_R (**Table 1**), the i.v. injection of B-9972 caused hypotensive responses that were not affected by pretreatment with enalaprilat, but significantly reduced by pretreatment with icatibant. Further, despite its diminished affinity for the B_2_R relative to BK, as judged from a binding assay to recombinant B_2_R conducted at 0°C in the presence of peptidase inhibitors (**Table 1**), the analog B-9972 is a more potent hypotensive agent than BK *in vivo* (lower threshold for effects; compare **Figures 2C** and **4B**); B-9972 is nearly as potent as BK tested in the presence of enalaprilat. Although we might have expected that the synthetic peptide B-9972 exhibits prolonged hypotensive effects (independently of potency) as inferred from previous studies involving infusion of B-9972 or comparably designed peptides resistant to peptidases (Taraseviciene-Stewart et al., 2005; Marketou et al., 2010; Potier et al., 2013), the hypotensive episodes were brief and had similar temporal profiles than those elicited by BK; except for the highest dose of B-9972, that was associated with a longer half-recovery time than BK. It is likely that the chosen administration route, i.v. boluses, leading to rapid dilution in the organism, was responsible for the brief hypotensive episodes noted with B-9972. Biphasic effects of B-9972 on HR were generally comparable to those of BK but of higher amplitudes at highest doses and a lower dose threshold for a significant effect (**Figure 3**).

Extending a recent *in vitro* study from this laboratory (Charest-Morin et al., 2014), we provide further pharmacological evidence of BK regeneration from extended BK sequences that behave as B_2_R agonists following a limited proteolysis. The basic postulate of this line of investigation is that the prolonged peptides have negligible affinity for the B_2_R, but can regenerate BK according to a precise cleavage rule (**Table 1**; **Figure 1**). Using the C-terminally extended peptide, BK-Arg, as a potential substrate of kininase I–type carboxypeptidases, we provided pharmacological evidence that BK-Arg behave as an Arg-carboxypeptidase-activated B_2_R agonist, as the hypotensive response to this analog was strongly inhibited by pretreatment with icatibant and the Plummer’s inhibitor, a mercapto analog of Arg (Plummer and Ryan, 1981) (**Figure 5**). These results further support previous findings from this laboratory showing a loss of BK-Arg contractile potency in a venous contractile bioassay in the presence of the Plummer’s inhibitor (Charest-Morin et al., 2014). Interestingly, the hypotensive response elicited by the highest dose of BK-Arg was potentiated in the presence of enalaprilat. Considering that BK-Arg has very little direct affinity for ACE (**Table 1**), it is thus plausible that the enhanced response was consecutive to a potentiation of regenerated BK.

Pharmacological evidence of B_2_R-mediated hypotensive response to BK-His-Leu or BK-Ala-Pro, two C-terminally extended BK peptides shown to have very little direct affinity at the B_2_R (Charest-Morin et al., 2014), was obtained as icatibant abated the hypotensive effect of each of these peptides. However, in contrast to what we previously found in the human umbilical vein contractility assay, and despite the good affinity of both peptides for ACE [**Table 1**; (Charest-Morin et al., 2014)], the simple postulated cleavage rule leading to BK regeneration following a single catalytic step mediated by ACE was not supported. Indeed, pretreatment with enalaprilat failed to reduce the hypotensive response of either prolonged peptide. When ACE is blocked, the gain of function resulting from the regeneration of BK *in vivo* must follow alternative cleavage rules involving unidentified carboxypeptidase(s). In turn, regenerated BK may be considerably potentiated by ACE blockade, leading to such paradoxical results as a gain of apparent potency of BK-Ala-Pro in response to enalaprilat pretreatment (**Figure 7**). Therefore, BK-His-Leu and BK-Ala-Pro are certainly pro-drugs that release BK, but in a more complex metabolic context than that anticipated from previous *in vitro* experiments.

The possible involvement of inducible B_1_R to the transient hypotensive response elicited by the three C-terminally extented analogs appears unlikely as these peptides were shown to have little or no direct affinity for the B_1_R (Charest-Morin et al., 2014). Although it is not excluded that the prodrug peptides indirectly produce small amounts of the B_1_R agonist des-Arg^9^-BK from regenerated BK or otherwise, we showed that the effects of des-Arg^9^-BK were extremely small in healthy rats (**Figure 1D**), in which the expression of inducible B_1_Rs should be minimal (Leeb-Lundberg et al., 2005).

Both pro-drug peptides BK-Arg and BK-His-Leu had a tendency to produce prolonged hypotensive episodes with, occasionally, a slow onset, which might result from a slow and progressive regeneration of BK following limited proteolysis (see for instance **Figure 8A**). However, this was variable and statistics on half-recovery time for hypotensive episodes generally failed to show statistical difference from those recorded following similar doses of BK under the applied protocol (bolus administration of peptides).

Using miniature pulsed Doppler flow probes acutely implanted in the experimental animals (**Figure 8**), we demonstrate that the transient hypotensive response to BK and all tested alternate kinin receptor agonists coincided with a concomitant increase in the mHDS signal and tachycardia. The increase in mean Doppler shift might reflect a change in vascular resistance (vasodilation) redistributing flow to the hindlimb muscles, as previously shown by one of us in conscious rats (Bachelard et al., 1992a). These vasodilator effects might likely involve the synthesis and release of endothelial relaxing factors after the activation of B_2_Rs, such as NO and prostaglandins (Nasjletti and Malik, 1979; D’Orléans-Juste et al., 1989; Veeravalli and Akula, 2004).

The present study constitutes an *in vivo* confirmation of the differential susceptibility of a set of BK analogs to peptidases, B-9972 being totally ACE-resistant and the pro-drug BK-Arg regenerating BK via the action of Arg-carboxypeptidase activity. Additional pro-drug peptides, BK-His-Leu and BK-Ala-Pro, also regenerate BK *in vivo*, probably by more than one cleavage rule. Whether peptidase-activated B_2_R agonists evade extra-vascular effects by progressively releasing the fragile peptide BK in the microcirculation remains to be established. Future studies may compare the burden of side effects (e.g., plasma extravasation) of the two general strategies, administration of a pro-peptide that releases BK vs. the use of a peptidase-resistant agonist like B-9972.

## Author Contributions

MJ performed the experiments, analyzed the data, and reviewed drafts of the paper. LG provided unique reagents, analyzed the data, and reviewed drafts of the paper. XC-M performed some experiments, designed some of the experiments and reviewed drafts of the paper. HB and FM conceived and designed the experiments, analyzed the data, wrote the paper, prepared figures and/or tables, reviewed drafts of the paper. All authors approved the version to be published.

## Conflict of Interest Statement

The authors declare that the research was conducted in the absence of any commercial or financial relationships that could be construed as a potential conflict of interest.
